# The Interplay Between Diet and the Epigenome in the Pathogenesis of Type-1 Diabetes

**DOI:** 10.3389/fnut.2020.612115

**Published:** 2021-01-28

**Authors:** Amira Kohil, Maha Al-Asmakh, Mashael Al-Shafai, Annalisa Terranegra

**Affiliations:** ^1^Department of Biomedical Sciences, College of Health Sciences, QU Health, Qatar University, Doha, Qatar; ^2^Biomedical Research Center, Qatar University, Doha, Qatar; ^3^Biomedical and Pharmaceutical Research Unit, QU Health, Qatar University, Doha, Qatar; ^4^Research Branch, Sidra Medicine, Doha, Qatar

**Keywords:** type 1 diabetes, diet, epigenetics, histone modifications, DNA methylation, micro-RNA

## Abstract

The autoimmune disease, Type 1 Diabetes Mellitus (T1DM), results in the destruction of pancreatic β-cells, and the International Diabetes Federation reports that its incidence is increasing worldwide. T1DM is a complex disease due to the interaction between genetic and environmental factors. Certain dietary patterns and nutrients are known to cause epigenetic modifications in physiological conditions and diseases. However, the interplay between diet and epigenetics is not yet well-understood in the context of T1DM. Several studies have described epigenetic mechanisms involved in the autoimmune reactions that destroy the β-cells, but few explored diet components as potential triggers for epigenetic modifications. Clarifying the link between diet and epigenome can provide new insights into the pathogenesis of T1DM, potentially leading to new diagnostic and therapeutic approaches. In this mini review, we shed light on the influence of the diet-epigenome axis on the pathophysiology of T1DM.

## Introduction

T1DM is a metabolic disease resulting from chronic autoimmune destruction of the insulin-producing pancreatic β-cells (1). It primarily affects children and adolescents and can lead to complications including ocular damage, stroke, diabetic ketoacidosis, coma, and kidney failure (2). The disease's incidence is increasing worldwide, with approximately one million cases annually [International Diabetes Federation (IDF) Diabetes Atlas 2014]. This may be due to recent advances in early diagnosis and monitoring of T1DM (1). However, the sedentary lifestyle predominant worldwide and especially in westernized countries has a strong impact toward developing autoimmune disorders, including T1DM (3). This lifestyle, characterized by a high-fat/low-fiber diet and lack of physical activity, is known to strongly modulate the immune system and can lead to T1DM primarily through its impact on T-cells. For example, in the United states, T1DM incidence increased by 21% among young adults (<20 years old) from 2001 to 2009 (4). Moreover, T1DM is highly prevalent among those <19 years of age in countries with a crescent economy and a shift toward a western-like lifestyle, as in the Middle East region, including Kuwait (44.5%), Saudi Arabia (33.5%), and Qatar (12.2%) (5). Similarly, in India the prevalence of T1DM is 31.9 per 100,000, with higher prevalence seen in urban areas compared to rural areas (6). In Korea between 2007 and 2013, the annual incidence of T1DM increased from 2.73 to 5.02 per 100,000 (7). These numbers illustrate the increasing worldwide prevalence of T1DM and indicate a noticeable impact in countries that have recently adopted a westernized lifestyle.

T1DM involves a complex interaction between genetic and environmental factors, and several genetic loci have been linked to the disease (8, 9). This includes strongly-associated genetic variants at the human leukocyte antigens DR and DQ (HLA-DR and -DQ) that confer a high risk for the development of T1DM (10). Variants in other autoantigens, such as those targeting pre-proinsulin, β-cell specific zinc transporter 8, insulinoma-associated antigen 2, the insulin gene, the cytotoxic T-lymphocyte–associated protein-4, and the interleukin-2 receptor are also known to contribute to the susceptibility of T1DM (9, 11, 12). However, genetic factors cannot explain all T1DM cases and sub-phenotypes. Many environmental factors have also been associated with greater risk of T1DM, including infections, dietary factors, old maternal age, psychological stress, use of antibiotics, mode of delivery, and steroids intake (13, 14). Diet and nutrients, among these factors, are known to exert a direct effect on the epigenome (15).

Epigenetic modifications are influenced by the interaction between genetics and the environmental stimuli, resulting in a T1DM specific epigenetic status (16, 17). Moreover, it is proposed that diet can lead to epigenetic changes and immune dysregulation in T1DM pathogenesis with mechanisms similar to what is seen in other inflammatory disorders (18). Since T1DM is a complex disorder a multivariate pathogenesis, a greater understanding of the interplay between diet and epigenetics could provide new avenues for early diagnosis, treatment, and a personalized therapy. This review aims to summarize the role of both diet and epigenome in T1DM development, as well as to clarify the interplay of diet-epigenome in the pathophysiology of T1DM.

## The Role of Diet in The Pathogenesis of T1DM

Diet is considered an environmental trigger involved in the development of various metabolic disorders, such as obesity, type 2 diabetes mellitus (T2DM), and T1DM (19–21). Some dietary factors such as fat, protein, and fibers are known to affect the glycemic control in T1DM patients, but the molecular mechanisms by which diet and nutrients impact the development of T1DM are still unknown. Below, we summarize the recent evidences on the role of nutrition in T1DM, focusing mainly on food habits and nutrients that can exert epigenetic changes.

### Early Nutrition

Since T1DM and its pre-clinical autoimmunity appear at an early age, several studies highlighted the role of early nutrition in the pathogeneses of T1DM and is also considered a risk factor of the disease. Dietary habits after birth are investigated as potential determinants of individual risk for developing T1DM. Breastfed children, for instance, have shown a lower risk of developing T1DM (22) due to the presence of secretory immunoglobin A, lysosomes, and lactoferrin in breast milk (23). In contrast, increased intake of cow's milk or formula milk, from 6 to 9 months of age, was suggested to increase the risk of developing T1DM (23, 24). This finding is likely, due to the presence of specific fatty acids (e.g., penta-decanoic, myristic, and isomers of monounsaturated palmitoleic acid), which may promote islet autoimmunity (25). Moreover, the intake of hydrolyzed formula (lacking of complex dietary proteins) has no effect on reducing the incidence of T1DM in infants at high risk (26). In addition, the early introduction of cereals that do or do not contain gluten into the diet may promote the development of T1DM, which can be attributed to the immature immune system and undeveloped gut microbiota present in young children, whereas late introduction (after 6 months) of gluten has no effect (27). However, contrasting data showed that delayed introduction of cereals containing gluten is associated with increased risk of developing islet autoimmunity and progression to T1DM (28). Furthermore, one study showed that serum fatty acids, biomarkers of milk, and ruminant meat consumption, are significantly associated with advanced β-cell autoimmunity in children with conferred susceptibility to T1DM compared to seronegative controls (19).

### Diet in Adult T1DM Patients

In adults with T1DM, a low-carbohydrate/high-fat diet has been associated with improved glycemic control, shown via low hemoglobin A1c (HbA1c) and less glycemic variability. However, this diet was also linked to an increased risk of hypoglycemia and dyslipidemia (29). In the case of fiber intake, one study showed that low intake of dietary fibers is associated with elevated levels of HbA1c in T1DM patients, thus leading to a poor glycemic control (30). On the other hand, another study showed that a high fiber diet is associated with decreased protein synthesis and degradation (post-absorptive protein turnover) in T1DM male patients (31). Nutritional intake was also found to influence the development of T1DM-related complications. A study conducted by Beretta et al. showed that a diet containing high fibers exhibited a significant reduction in BMI, systolic/diastolic blood pressure, and reduction in energy intake compared to T1DM patients with a low fiber diet (32) development of T1DM-related complications has also been linked to nutritional intake. The intake of complex carbohydrates was linked to the presence of diabetic retinopathy in T1DM adult patients, whereas the intake of unsaturated fat, particularly monounsaturated fatty acids (MUFA), was associated with the absence of diabetes retinopathy (33). In another study, a positive correlation was observed between high intake of saturated fats and proteins and the risk of developing coronary heart disease (CHD) in T1DM patients, while high carbohydrate intake was negatively correlated with CHD (34). Vitamin D also plays important role in regulating both the immune system and metabolic pathways, and several studies have demonstrated that vitamin D supplementation lowers the risk of developing T1DM (35, 36). This effect could be explained by the observation that vitamin D down-regulates the response of T helper-1 lymphocytes (37). However, other investigations did not confirm such effect of vitamin D supplementation (38, 39). These discrepancies could be attributed to the differences in the type of supplement (i.e., cholecalciferol, alpha-calcidol, or calcitriol), the vitamin dosage, the age group of the study participants, and/or the duration of diabetes.

## The Role of Epigenetics in T1DM Pathogenesis

Epigenetics is the activation and inhibition of gene expression by factors that does not affect the DNA sequence itself (40). Such epigenetic changes are affected by genetic variations as well as environmental factors and influencing DNA accessibility by transcription factors leading to the regulation of gene expression (16, 17). It has been shown that epigenetic modifications, mainly DNA methylation, microRNAs, and histone modifications, play an important role in developing various autoimmune disorders, including T1DM (41, 42). Several features of T1DM support the involvement of epigenetics in its pathogenesis. These include high discordance rates among monozygotic (MZ) twins, offspring of an affected father rather than affected mother having a higher risk of developing the disease, and the increased T1DM incidence in genetically stable populations (41, 42) and countries with a westernized lifestyle (3).

### DNA Methylation Modifications in T1DM

DNA methylation is defined as the formation of 5-methylcytosine (5-mC) by the attachment of a methyl group on the fifth carbon of cytosine, and it usually occurs in the CpG dinucleotides (43). Three methyltransferases - DNMT1, DNMT3a, and DNMT3b - are primarily responsible for regulating DNA methylation process, which are DNMT1, DNMT3a, and DNMT3b (44). The main function of DNMT1 is to maintain the methylation status in the genome, restoring the methylation pattern in the daughter strands during replication. DNMT3a and DNMT3b, conversely, are responsible for establishing de novo DNA methylation patterns (44). DNA demethylation on the contrary, is a sequential oxidation of 5-mC and the removal of the modified group by thymine DNA glycosylase (TDG) produce cytosine in replacement of 5-mC. The demethylation process also involves Ten-eleven translocation (TET) family dioxygenase enzymes, which are TET1, TET2, and TET3 (45, 46). DNA methylation has been implicated in a number of autoimmune disorders, including T1DM (47). Alterations in DNA methylation may cause changes in the expression of genes responsible for β-cell survival, insulin secretion, and autoimmunity, thereby influencing the development of T1DM (42). This hypothesis is supported by research from Rakyan and colleagues, who generated a genome-wide methylation profile from T1DM-discordant MZ twins. They identified 132 T1DM-associated methylation sites in the promoter regions of genes associated with apoptosis, inflammation, and immune regulation. Additional evidence comes from Stefan and colleagues, who detected 88 differentially-methylated CpG sites in T1DM-discordant MZ twin pairs (48). Although, this is an observational study, the enrollment of MZ twins as study subjects reinforces the value of the findings, providing a strong evidence that DNA methylation plays an integral part in T1DM development and thus not totally explained by genetics.

The INS gene locus is closely involved with T1DM, and its A/T single nucleotide polymorphism (SNP) rs689 in particular has been associated with T1DM development (8). A number of studies found DNA methylation of INS gene promoter in pancreatic β-cells and thymic epithelial cells is significantly implicated in T1DM development (49, 50). One study showed that patients with T1DM have a higher level of methylation at CpG−180 and lower level at methylation of CpG−19,−135, and−234 in INS gene when compared to healthy controls (49). They also found a strong correlation between high methylation levels of CpG −69, −102, −180, −206, and T1DM. However, it is unknown whether these epigenetic changes in T1DM precedes or follows the development of the disease (49). In addition, a study conducted by Rui et al. found that INS gene expression is regulated by the methylation of Ins1 exon-2 and Ins2 exon-1. The study showed, both in NOD mice (*in-vivo*) and in human β-cells (*in-vitro*), that pro-inflammatory cytokines could activate methyltransferases, leading to methylation of Ins1 exon-2 and Ins2 exon-1 in the INS genes (50).

Interleukin 2 receptor α-chain gene (IL2RA) encodes the IL-2 receptor, which is highly expressed in regulatory T-cells Tregs and plays a vital role in suppressing autoreactive T-cells. Like the INS gene, epigenetic modulation of the IL2RA gene was implicated in the development of T1DM. One study showed that T1DM patients have a high methylation level at IL2RA CpGs −373 and −456 compared to healthy controls (51). They found that the DNA methylation at IL2RA CpGs −373 was associated with 16 known SNPs to be involved in T1DM.

DNA methylation was also implicated in the complications associated with T1DM, such as diabetic nephropathy (52). Methylation of 19 CpG sites was correlated with the development and onset of diabetic nephropathy. Furthermore, one of the methylated CpG sites was located near the transcription start site of UNC13B (rs13293564), which is known to be involved the development of diabetic nephropathy in T1DM patients (52).

Finally, it is worth noting that epigenetic mechanisms are also implicated in the early development and function of the insulin producing pancreatic β-cells. Different types of epigenetic modifications were found to be involved in the development of pancreatic cells from the endocrine progenitor cells (53). Particularly, a study conducted by Neiman et al. found that promoters of β-cell specific genes, such as Insulin 2 (INS2) and Glucagon genes, were significantly hypermethylated in non-endocrine tissues when compared to β-cells (54). The same study also found different methylation levels of the CpG sites amongst the endocrine cells' subtypes (α-cells, β-cells, and δ-cells), that may implicate a specific control of the gene expression in these cells (54).

### The Micro-RNA Modifications in T1DM

The micro-RNA (miRNA) are non-coding RNA molecules ranging from 18 to 22 nucleotides that act as post-transcriptional silencers (55), and are involved in several biological processes, such as cell division, proliferation, and apoptosis (56). In the nucleus, the miRNA is transcribed into primary miRNA by RNA polymerase (RNase) II and III and then processed to precursor miRNA by the complex Drosha/DGCR8. The precursor miRNA is finally processed into the mature miRNA in the cytoplasm by RNase III Dicer complex (57). miRNAs exert their post-transcriptional effect through binding to the 3′ untranslated region of the targeted mRNA, leading to its cleavage, degradation and translation inhibition (58). The effect of miRNAs on gene expression was implicated in different autoimmune disorders, such as autoimmune thyroid diseases (59) and rheumatoid arthritis (60). In the case of T1DM, alterations in miRNA levels may contribute to the disease pathogenesis by affecting multiple pathways, such as insulin secretion, the programmed cell death, the immune system, and the mitogen-activated protein kinase (MAPK) signaling pathway (61). A group of miRNAs (miR-210-3p, miR-155-5p, miR-103a-3p, and miR-146a-5p) was identified as differentially regulated in T1DM, and it was mainly associated with immune system functions (61). Several studies identified the mechanism of single small groups of miRNAs. To mention some, Krishnan et al. found that upregulation of miR-155-5p in human islet derived exosomes targets the mRNA of the transcriptional and immune response regulator gene (TCIM) (62). Another study by Garcia-Diaz et al., found that the upregulation of miR-155-5p has a role in the inflammatory process in T1DM through binding to the toll-like receptors and activating the NF-κB pathway (63). A more recent study identified a different set of miRNAs (miR-142-5p, miR-146a-5p, and miR-223-3p) positively correlated with B lymphocyte, CD4^+^CD45RO^+^, and CD4^+^CD25^+^ immune cells in T1DM patients (64). Low expression levels of miR-146a-5p were consistently observed in T1DM patients, and it was associated with overproduction of IL-6, which is an important pro-inflammatory cytokine. Therefore, it could be suggested that miR-146a-5p has a negative feedback effect on the NF-κB pathway in regulating the inflammation status in T1DM (63, 65). In addition, the overexpression of miR-23b, miR-98, and miR-590-5p in CD8^+^ T-cells from T1DM patients was suggested to target apoptotic genes (TRAIL, TRAIL-R2, FAS, and FASLG), resulting in the excessive proliferation of autoreactive T-cell and T1DM development (66). Furthermore, hyperglycemia was found to influence the levels of certain miRNAs, such as miR-125b-5p and miR-365a-3p that were positively correlated with HbA1c levels (67). Circulatory miRNAs can also be used as a biomarker for early detection of T1DM as they are stable and easily detected (61). This was supported by a variety of studies; for example, miR-125-5p and miR-320c were identified in early onset-T1DM patients and were elevated before progression of diabetes in NOD mice (68). Furthermore, urinary miR-377 was found to be positively correlated with HbA1c and urinary albumin creatinine ratio in T1DM patients, which makes it a possible biomarker for diabetic nephropathy (69). Identifying early miRNA biomarkers may help in early diagnosis, treatment and prevention of diabetic complication.

### Histone Modifications in T1DM

Histones undergo different modifications, such as methylation, acetylation, phosphorylation, and other mechanisms (sumoylation and ubiquitination) in specific amino acid residues located in the N-terminal part (70). The histone methylation involves the addition of methyl groups to lysine or arginine residues, resulting in activation or inhibition of transcription based on the level of modification and the affected region (71), while histone demethylation involve the binding of a lysine specific demethylase-1 (LSD1) to the lysine methylation site on the histone tail, removing the methyl group (72). The histone acetylation involves the addition or removal of acetyl group on lysine residues by the function of histone-acetyltransferases (HATs) and histone-deacetylases (HDACs), respectively (17). Histone acetylation promotes an opened chromatin structure that is more accessible for gene transcription by reducing the electrostatic affinity between protein and DNA (73). Histone ubiquitination, is defined as the addition of ubiquitin molecules to the conserved lysine residues through the function of ubiquitin ligases (74). Furthermore, sumoylation is defined by the attachment of ubiquitin like modifier proteins covalently to histones through the action of ubiquitin analog enzymes (75). Histone modification has been associated with different pathological conditions, including T1DM. One study found a significant increase of H3 lysine-9 di-methylation (H3K9me2) in T1DM patients' lymphocytes compared to healthy controls (76). They also found a strong association between increased H3K9me2 promoter activity in CLTA4 gene (T1DM susceptibility gene) and genes involved in autoimmune and inflammatory pathways, such as TLR, NF-κB, and p38-MAPK (76). Furthermore, T1DM patients were found to exhibit marked variations in H3 lysine-9 acetylation (H3K9Ac) levels at the upstream regions of HLA-DRB1 and HLA-DQB1, which are susceptibility loci strongly associated with T1DM (77). The same study further demonstrated that THP-1 monocytes treated with interferon-γ and TNF-α showed increased expression of HLA-DRB1 and HLA-DQB1 combined with changes in H3K9Ac, similarly to what was observed in T1DM patients (77). Moreover, another study showed an increased level of H4 acetylation in T1DM patients, however it was restricted to T1DM patients with no cardiovascular complications, indicating that histone acetylation may have a protective role against the development of T1DM complications (78). More studies are needed to clarify the mechanism of histone modifications affecting T1DM susceptibility loci and their impact on the development of T1DM.

## Diet and Epigenetic Interplay in T1DM Pathogenesis

The interplay between diet and epigenetics and how this link contributes to the pathogenesis of T1DM is yet to be identified. In this review, we suggest that dietary habits as well as specific nutrients may result in certain epigenetic signatures contributing to T1DM development.

In general, three possibilities have been postulated on how nutritional factors modulate DNA methylation; first, by directly providing the substrates needed in the methylation process (methyl-donor nutrients, e.g., methionine, choline, and folate); second, by providing co-factors needed for the function of methyltransferases (e.g., vitamins B2, B6, and B12); third, by altering the activity of the enzymes involved in the methylation process [e.g., polyphenols; (79)].

Most of the dietary factors known to be involved in the pathogenesis of T1DM, such as breastmilk, fibers, MUFA, vitamin D, etc., are also known to have an epigenetic function (80–83), but their mechanism of action is not fully understood in the context of T1DM. We reviewed the major studies identifying a mechanism or a potential link of diet with DNA-methylation, histones modifications and miRNA in T1DM (Table 1).

**Table 1:** The interplay between diet and epigenetic modifications in different metabolic conditions.

**Epigenetic modification**	**Diet**	**Affected genes and pathways**	**Study population/experimental model**	**Metabolic disorder**	**References**
DNA methylation	High fat diet	Aberrant methylation at the promoter region of the TCF7L2 in β-cells.	Mice (male C57BL6J)	β-cells survival	(84)
	High fat diet	2-folds increase of 5 hmC (active demethylation of 5 mC) of the Rac1 promoter in T2DM mice.	Sprague Dawley rats (Male)	T2DM	(85)
	Maternal high fat diet	-Hypermethylation of insulin receptor substrate-2 gene (IRS-2). -Hypomethylation of mitogen activated protein kinase kinase-4 (MAP2K4).	Pups from female mice (C57BL6J)	Diabetes	(86)
	High carbohydrate diet	Differentially methylated genes are involved in glucose metabolism, insulin signaling, and lipid metabolism.	Ctenopharyngodon idellus	–	(87)
	GLP-1 agonist and nutritional consultation	An increase in the FFAR3 receptor methylation level in the obese group upon the nutritional counseling intervention.	Diabetic and obese patients	Diabetes and Obesity	(88)
	Low folate intake	-Low methylation levels of CAMKK2, thus increased gene expression. -Low methylation level of CAMKK2 is negatively correlated with insulin resistance	Obese patients	Obesity	(89)
	Folic supplementation	Differential methylation in 3,787 genes involved in insulin secretion and pancreas function; improved insulin resistance and fat reduction.	Mice (male C57BL6J) treated with high fat diet	Obesity	(90)
	Vitamin B12 supplementation	-589 differentially methylated CpG and 2,892 differentially methylated regions, mostly hypomethylated. -Significant enrichment in T2DM genes and pathways. -miR21 methylation that inhibits its function and downstream T2DM related genes.	Children with vitamin B12 deficiency	Vitamin B12 deficiency	(91)
miRNA	High fat diet	Elevated expression of miR-495, and consequent transformation of macrophage M2 in to the pro-inflammatory M1 and increased insulin resistance.	Mice (male C57BL6J)	T2DM	(92)
	High fat diet	Enhanced expression of miR-122 with downregulation of the insulin-like growth factor 1 receptor (IGF-1R).	Sprague Dawley rats (Male)	Diabetes	(93)
	High protein and fish oil	Downregulation of miRNAs (miRs-411,−155,−335, and−21), involved in inflammation, dyslipidemia, and hyperglycemia.	Mice (NZ10 and SWR/J)	Obesity/Diabetes	(94)
	High fat diet	Overexpression of miR-125a improving insulin sensitivity and preventing hepatic lipid accumulation by targeting ELOVL6 gene.	Mice (Male C57BL/6 and ob/ob mice)	Obesity	(95)
	Flavonoids (isoliquiritigenin/liquiritigenin)	-Inhibition of miR-122 via inhibition of JNK pathway and restoring the function of IRS1/IRS2 and insulin signaling. -Abrogated the insulin resistance observed in mice under high fat diet.	Mice (Male C57BL/6)	Obesity induced insulin resistance	(96)
	LC-PUFA	Downregulation of 5-lipoxygenases through miR-146a-5p, which targets pathways related to TGF-β, ECM-receptor signaling, fatty acid and steroid biosynthesis.	Mice (Female C57BL/6)	Allergic Asthma	(97)
Histone modification	Maternal fat diet	Significant decrease in the level of both H3K9Ac and H3K9me1; and increase in H3K4me3	Mice (C57BL/6)	Obesity	(98)
	High amylose maze starch acetylated (HAMSA)/or butyrylated (HAMSB)	HAMSB diet increased the abundance of acetylated H3K9 and H4 at the Foxp3 promotor in T-cells.	Mice (NOD/Lt, C57BL/6 and NOD 8.3)	T1DM	(99)
	Resveratrol	-Decreased expression of CCR6 gene, encoding the chemokine c-c motif receptor. -Reduced levels of CCR6+ IL-17-producing cells and CD11b^+^F4/80^hi^ macrophages in spleen and pancreatic cells.	NOD Mice (H-2G7 and BDC2.5)	T1DM	(100)

Cow's milk allergenicity is considered one of the dietary risk factors for T1DM, as shown in a study where A1 beta-casein from cow milk was associated with an increased T1DM incidence and sub-clinical insulitis across generations (101). The authors suggested that beta-casomorphin peptide (BCM-7) released from A1 beta-casein may cause epigenetic alterations that lead to T1DM development. In gastrointestinal diseases, BCM-7 was found to act as a epigenetic modulator and to differentially methylate genes involved in these diseases (102). It is therefore speculate that the beta-casein effect observed in T1DM can cause differential DNA methylation of similar or other genes.

High intake of fat was reported to be associated with risk of islet autoimmunity, poor glycemic control, and development of T1DM-related complications during both infancy and adulthood in T1DM patients (34, 103). High fat diet was found to epigenetically impact TCF7L2, a transcription factor required for pancreatic β-cell survival (84). An experimental study demonstrated that mice treated with high fat diet showed an aberrant methylation at the promoter region of the TCF7L2 in β-cells compared to mice fed with a normal diet (84). Although defects in TCF7L2 were commonly detected in T2DM patients, a subset of non-obese T1DM patients were also found to have the same defect (104). Another animal study using a high fat induced hyperglycemic rats showed epigenetic involvement in T2DM animals (85). The study showed a significant 2-folds increase of 5 hmC (active demethylation of 5 mC) of the Rac1 promoter in high fat induced T2DM compared to healthy animals and compared to T1DM animals fed on normal diet. Moreover, high intake of saturated fatty acid was associated with low levels of adiponectin in T1DM patients (105) and several studies showed that dietary fatty acids regulate adipocyte function through epigenetic modifications, mainly polyunsaturated fatty acids (PUFA) and saturated fatty acids (86, 106, 107). In a similar human study, the high intake of saturated fatty acids and PUFA was associated with increased DNA methylation level in the adipose tissue, which was correlated with increase in body weight (106). A total of 1,797 genes were deferentially methylated under high PUFA, whereas 125 genes were deferentially methylated in the case of saturated fatty acid intake. An animal-based study found that maternal high fat diet leads to hypermethylation of insulin receptor substrate-2 gene (IRS-2) and hypomethylation of mitogen-activated protein kinase kinase-4 gene (MAP2K4) in mice offspring; thus, decreasing and increasing the gene expression, respectively, and elevating pup's risk of developing diabetes in the future (86). Maternal high fat diet has negative effects on the pancreatic β-cells of male mice offspring, in which proliferation defects and insulin degranulation were detected (107). However, this phenotype was reversed and offspring were protected from developing insulin resistance through early transition from high fat diet to normal fat diet (107).

The diet-epigenetic axis is also implicated in pathways related to glucose metabolism and insulin signaling, as demonstrated in animal models. Whole genome DNA methylation analysis of the grass carp, Ctenopharyngodon idellus, revealed no significant changes in methylation level of metabolic genes between different nutritional conditions (high carbohydrate diet/ normal diet) (87). However, the deferentially methylated genes in Ctenopharyngodon idellus with a high carbohydrate diet were enriched in pathways involved in glucose metabolism, insulin signaling, and lipid metabolism. Most of these obesity and T2DM- involved methylation changes were also observed in mammals (87). Moreover, folic acid (synthetic form of folate) is mainly involved in the methylation processes acting as a methyl donor for the synthetic process of S-adenosylmethionine (108). Low folate intake was associated with the methylation of Calcium/Calmodulin Dependent Protein Kinase Kinase 2 (CAMKK2), which is an important gene involved in glucose homeostasis and adiposity. Obese patients with low folate intake, showed lower methylation levels of CAMKK2, a condition correlated with increased insulin resistance (89). A study conducted by Li and his colleagues found that folate supplementation in mice treated with high fat diet reduced fat mass and improved insulin resistance (90). They also found that deferentially methylated regions associated with folic supplementation mainly affected adipose genes and were involved in pathways related to insulin secretion and pancreatic function. Vitamin B12 also found to be involved in the synthesis of S-adenosylmethionine through acting as a coenzyme that catalyzes the methylation of homocysteine to methionine, supporting its essential role in the methylation process (109). A recent study detected 589 differentially methylated CpG upon vitamin B12 supplementation, in which 73.3% of the genes were hypomethylated (91). In the same study, pathway analysis revealed significant enrichment in pathways related to T2DM, such as glycogen synthesis and adipogenesis pathways. Among the identified genes that were differentially methylated, the authors focused on the differential methylation of miR21, which is known to target different genes involved in T2DM. Their analysis found that miR21 was repressed upon vitamin B12 supplementation leading to inhibiting of its targeted genes (FTO, TCF7L2, CREBBP/CBP, and SIRT1) (91).

There is presently a lack of studies that demonstrate an interaction between diet and miRNA levels in T1DM. However, there are a number of studies that show this correlation in other metabolic disorders, such as in T2DM and obesity [Table 1; (92–95)]. An animal-based study found that mice given high fat diet showed elevated expression of miR-495, which caused the transformation of macrophage M2 into the pro-inflammatory M1 and increased insulin resistance (92). This study has showed the possible mechanism of miR-495 that acts as a negative regulator to the FTO gene and induces adipose tissue inflammation in T2DM mice given high fat intake. Another study showed enhanced expression of miR-122 accompanied with downregulation of insulin-like growth factor 1 receptor (IGF-1R) in the liver of diabetic rats given high fat diet (93). Furthermore, in mice susceptible of developing obesity and diabetes, a diet rich with protein and fish oil has been found to override this genetic susceptibility (94). It has been shown that certain miRNAs (miRs-411, 155, 335, and 21) involved in inflammation, dyslipidemia, and hyperglycemia were downregulated in mice fed with high protein and fish oil diet (94). A study conducted by Liu and colleagues showed that miR-125a level was downregulated in both genetic and dietary (high fat diet) mouse model of obesity (95). In obese mice treated with high fat diet, overexpression of miR-125a improved insulin sensitivity and prevented hepatic lipid accumulation by targeting Elongation of very long chain fatty acids protein 6 (ELOVL6) gene, which is a microsomal enzyme that catalysis the elongation of both saturated fatty acid and MUFA (95). In addition, long chain PUFA (LC-PUFA) was found to modulate miRNA expression in a murine allergic asthma model, where it plays a protective role in the inflammatory process associated with asthma (97). After LC-PUFA supplementation, 21 out of 62 dysregulated miRNAs in asthmatic mice were restored, in which some of the restored miRNAs (mainly miR-146a-5p) are implicated in the function of TGF-β, ECM-receptor signaling, fatty acid and steroid biosynthesis. Mechanistically, the study found that LC-PUFA downregulates 5-lipoxygenases through modulating the expression of miR-146a-5p; thus, acting as an epigenetic regulator. However, it was also found that LC-PUFA downregulates 5-lipoxygenases independent of miR-146a-5p (97). Furthermore, in obese mice fed a high fat diet, flavonoids were shown to inhibit miR-122 dysregulation through the inhibition of JNK, thereby restoring the function of IRS1/IRS2 tyrosine phosphorylation and insulin signaling (96).

The correlation between diet and histone modification in T1DM development is yet to be identified, but several studies were conducted in other metabolic disorders (Table 1). In T1DM, NOD mice treated with resveratrol, a polyphenol known to enhance the activity of sirtuin 1 (NAD dependent histone deacetylase), showed a reduced expression of CCR6 gene, responsible for encoding the chemokine c-c motif receptor (100). It also reduced the level of CCR6^+^ IL-17-producing cells and CD11b^+^F4/80^hi^ macrophages in spleen and pancreatic cells (100). One study has identified 21 core histone marks with at least 1.5-folds change in a prediabetic animal model of high-fat diet induced obese mice (110). Diet and histone modification interplay was also implicated in maternal obesity and diabetes, where appropriate fatty acid intake during gestation in mice were found to help offspring to cope with obesogenic conditions (98). In obesity resistant mouse model, a significant decrease was observed in the level of both H3K9Ac and H3K9me1 compared to control mice. However, no significant change was observed of histone markers between obesity resistant mice and obesity prone mice (high fat diet/obesogenic diet), a finding potentially due to the effects of their different diets. In addition, level of H3K4me3 was elevated in both obesity mice models in comparison to healthy controls (98).

The interaction between diet and epigenetics in T1DM pathogenesis could involve the gut microbiota. Specific dietary factors that potentially influence T1DM, including breastfeeding, high fiber intake, and low-fat diet, affect the composition of the gut microbiota and their metabolites (111, 112). Dietary fibers also affect the gut microbial composition as they are digested solely by these organisms, primarily the lower gastrointestinal tract (113). Fermentation of fibers by the gut microbiota produces short-chain fatty acids (SCFA), contributing to gut microbiota diversity (114). These SCFA, such as butyrate, acetate, and propionate, will lead to the specific activation of free fatty acid receptors 2/3 (FFAR2 and FFAR3). This activation inhibits histone deacetylase, leading to inhibition of the inflammatory cascade and the activation of Tregs (18). Administration of dietary fibers to non-obese diabetic (NOD) mice elevated their levels of SCFA, reduced the levels of inflammatory mediators, enhanced the integrity of the gut barrier, and activated Tregs, thereby reducing the incidence of T1DM (99). In patients with T2DM, SCFAs are involved in DNA methylation and histone modifications (88). More specifically, butyrate inhibits histone deacetylases, resulting in inhibition of NF-κB (115) and activation of the MAPK and ERK pathways (116) in intestinal Tregs, thereby down-regulating the pro-inflammatory cascade (117). In addition, in T2DM patients, binding of SCFA to the promoter region of FFAR3 reduces methylation of the CpG islands (88). Since some of the genetic and environmental factors associated with T1DM and T2DM are similar, studies should be designed to identify potentially similar underlying epigenetic mechanisms.

**Figure 1:**
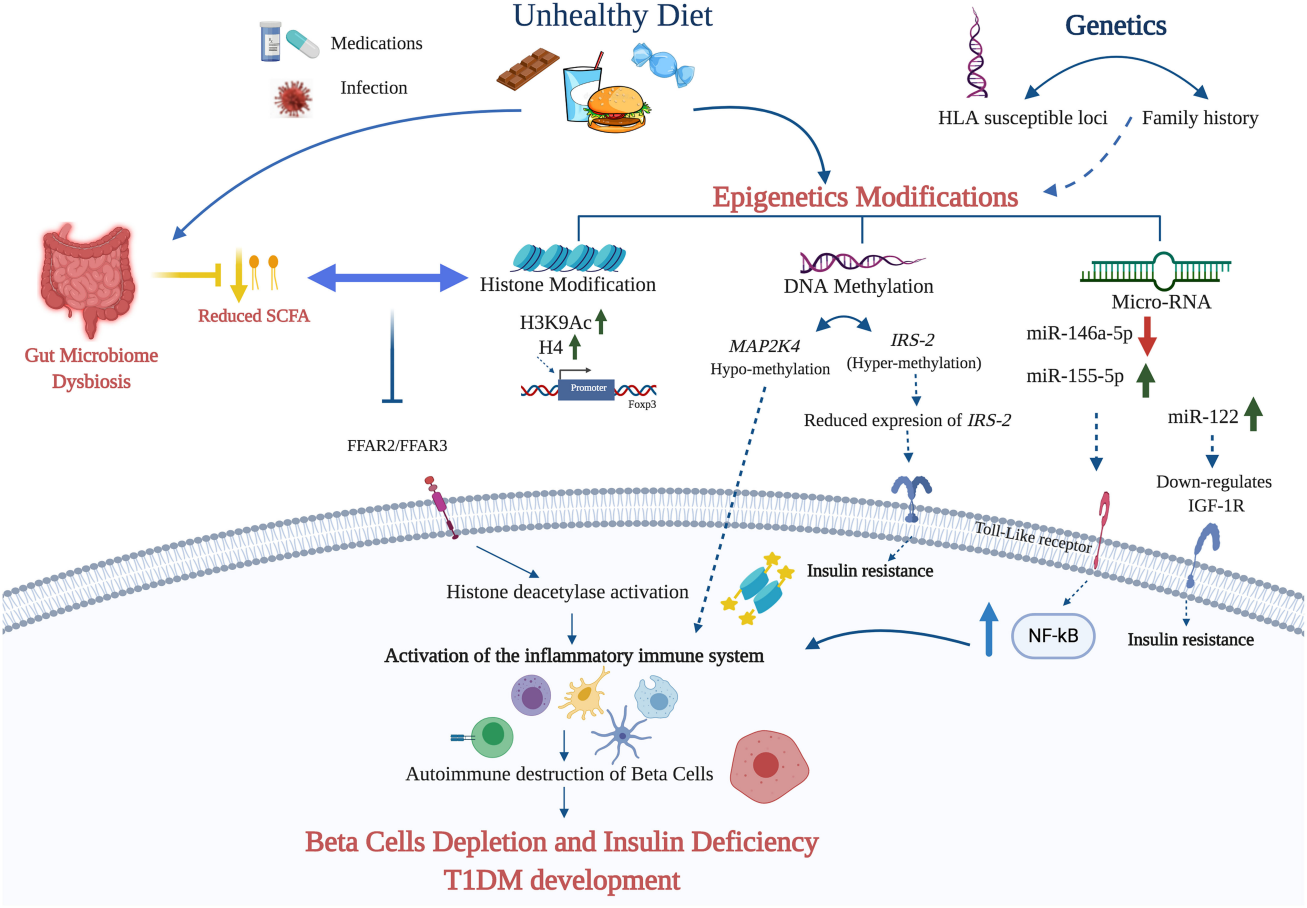
The figure summarizes the potential diet—epigenetic axis in T1DM development. The intake of unhealthy diet may lead to T1DM development through epigenetic modifications (DNA methylation, miRNA, and histone modifications). Diet nutrients can contribute to the hypermethylation and subsequent downregulation of IRS-2 leading to insulin deficiency. In addition, diet can contribute to the up- and down-regulation of certain miRNAs activates NF-kB pathway and the activation of the inflammatory response. The role of histone modification in T1DM is explicated mainly through elevating the expression of H3K9Ac and H4 in the promoter region of Foxp3. Finally, unhealthy diet affects the gut microbiota and its metabolites, such as SCFA. The failure of SCFA binding to the free fatty acid receptors 2/3 (FFAR2 and FFAR3) due to its absence or reduction, leads to the activation of histone deacetylase (HDAC1, HDAC3) and the inflammatory cascade that contribute to β-cell depletion and T1DM development (Created using Biorender.com).

Although epigenetic factors can affect the immune system directly in a manner that leads to T1DM (118), the molecular mechanism(s) underlying these epigenetic changes remain unclear. Our hypothesis is that, as in the case of T2DM and other metabolic conditions, different dietary factors contribute to the pathogenesis of T1DM by inducing epigenetic modifications directly or through the involvement of the gut microbiome [Table 1 and Figure 1; (18, 119)].

## Conclusions

T1DM is a complex disease caused by the interaction of many factors, both genetic and environmental, including epigenetics and dietary factors. Specific dietary patterns and nutrients can exert a direct impact on the immunopathogenesis of T1DM through epigenetic modifications. A deeper understanding of the interplay between the diet and epigenetics will improve our knowledge concerning the pathogenesis of T1DM and it will help identifying new therapeutic targets. We propose here a mechanism by which nutrients can trigger epigenetic modifications leading to β-cells depletion and T1DM development (Figure 1). A better characterization of the specific dietary patterns and nutrients that can exert such effects may help prevent and/or ameliorate T1DM and applying personalized nutrition approaches.

## Author Contributions

AT and AK developed the content of the manuscript. AK drafted the manuscript. AT, MA-A, and MA-S discussed the content and reviewed the manuscript. All authors contributed to the article and approved the submitted version.

## Conflict of Interest

The authors declare that the research was conducted in the absence of any commercial or financial relationships that could be construed as a potential conflict of interest.
